# Comparison of Blood Bacterial Communities in Periodontal Health and Periodontal Disease

**DOI:** 10.3389/fcimb.2020.577485

**Published:** 2021-01-05

**Authors:** David C. Emery, Tanya L. Cerajewska, Joon Seong, Maria Davies, Alex Paterson, Shelley J. Allen-Birt, Nicola X. West

**Affiliations:** ^1^Bristol Medical School, Translational Health Sciences, Learning & Research, Southmead Hospital, Bristol, United Kingdom; ^2^Periodontology, Bristol Dental School, University of Bristol, Bristol, United Kingdom; ^3^University of Bristol Genomics Facility, School of Biological Sciences, Bristol, United Kingdom

**Keywords:** bacteraemia, oral bacteria, periodontitis, MolYsis, inflammatory diseases

## Abstract

The use of Next Generation Sequencing (NGS) techniques has generated a wide variety of blood microbiome data. Due to the large variation in bacterial DNA profiles between studies and the likely high concentrations of cell-free bacterial DNA in the blood, it is still not clear how such microbiome data relates to viable microbiota. For these reasons much remains to be understood about the true nature of any possible healthy blood microbiota and of bacteraemic events associated with disease. The gut, reproductive tracts, skin, and oral cavity are all likely sources of blood-borne bacteria. Oral bacteria, especially those associated with periodontal diseases, are also commonly associated with cardiovascular diseases such as infective endocarditis, and also have been linked to rheumatoid arthritis and Alzheimer’s disease. Periodontal treatment, dental probing, and toothbrushing have been shown to cause transient bacteraemia and oral bacteria from the phyla Firmicutes (e.g. *Streptococci*) and Bacteroidetes (e.g. *Porphyromonas*) are found in cardiovascular lesions (CVD). Many studies of blood bacterial DNA content however, find Proteobacteria DNA to be the dominant microbiome component, suggesting a gut origin. Most studies of this type use total DNA extracted from either whole blood or blood fractions, such as buffy coat. Here, using a method that purifies DNA from intact bacterial cells only, we examined blood donated by those with active, severe periodontitis and periodontally healthy controls and show that 43–52% of bacterial species in blood are classified as oral. Firmicutes, consisting largely of members of the *Streptococcus mitis* group and *Staphylococcus epidermidis*, were predominant at 63.5% of all bacterial sequences detected in periodontal health and, little changed at 66.7% in periodontitis. Compared to studies using total DNA Proteobacteria were found here at relatively low levels in blood at 13.3% in periodontitis and 17.6% in health. This study reveals significant phylogenetic differences in blood bacterial population profiles when comparing periodontal health to periodontal disease cohorts.

## Introduction

Periodontal diseases, ranging from gingivitis, a reversible inflammation of the gingival margin, to severe periodontitis resulting in chronic inflammation of the tooth-supporting tissues with eventual bone and tooth loss, are the result of the accumulation of bacterial populations in dental plaque adjacent to soft tissue (Pihlstrom et al., 2005). Culturing techniques alone have identified over 500 species within the periodontal sub-gingival microbiota (Moore and Moore, 1994) with 16S ribosomal RNA (16S rRNA) gene polymerase chain reaction (PCR) studies revealing even richer populations (Kroes et al., 1999; Hajishengallis and Lamont, 2012). Within these populations there are conserved patterns of inter-species association (Gmur et al., 1989; Simonson et al., 1992; Ali et al., 1994) that follow a step-wise hierarchal biofilm development (Kilian et al., 2016). Many virulence factors are involved in biofilm organisation, host tissue penetration and host defence subversion (Holt et al., 1999). The resulting host inflammatory response to tooth-borne plaque, adjacent to soft supporting tissue, including vasodilation and increased permeability of blood vessels (Preshaw et al., 2004) facilitates the transfer of biofilm bacterial components into the bloodstream *via* the damaged and inflamed periodontium (Hajishengallis et al., 2011; Hajishengallis and Lamont, 2012; Hajishengallis, 2015). *Porphyromonas gingivalis (P.gingivalis)*, with many of its pathogenic functions mediated by gingipains (cysteine proteases), is central in this process (Hajishengallis et al., 2013; Maekawa et al., 2014).

Bacteraemia, as a direct result of periodontitis, has been proposed as a major contributory factor to pathology of most cardiovascular diseases (CVD) (Ross, 1999; Wilson et al., 2007; Lockhart et al., 2009; Tomas et al., 2012; Dietrich et al., 2013; Gargano and Hughes, 2014; Winning et al., 2015; Bale et al., 2017). Several oral bacterial species such as *Aggregatibacter actinomycetemcomitans (A. actinomycetemcomitans), P. gingivalis, Treponema denticola (T. denticola), Prevotella intermedia (P. intermedia)*, and *Tannerella forsythia (T. forsythia*), *Streptococcus mutans (S. mutans), Streptococcus sanguinis (S. sanguinis)* have all been detected in CVD lesions (Ford et al., 2005; Kozarov et al., 2005; Gaetti-Jardim et al., 2009; Leishman et al., 2010; Rafferty et al., 2011; Mahendra et al., 2013; Armingohar et al., 2014). Streptococcal species of the *viridans* group, containing the *Streptococcus mitis* (*S. mitis*) group are the taxa most commonly associated with community-acquired native valve infective endocarditis. Periodontitis, oral pathogens and the dysbiotic oral microbiome have also been linked to the aetiology of other inflammatory diseases such as rheumatoid arthritis (Loyola-Rodriguez et al., 2010; Zhang et al., 2015; Konig et al., 2016; Beyer et al., 2018; Correa et al., 2019; Gomez-Banuelos et al., 2019; Hammad et al., 2019), late onset Alzheimer’s disease (Singhrao and Olsen, 2019) and type II diabetes, which has been linked to distinct microbiomes in the gut (Wellen and Hotamisligil, 2005; Larsen et al., 2010), blood (Qiu et al., 2019), and oral cavity (Long et al., 2017).

Early studies of bacteraemia associated with oral activity focused on the transient effects of daily oral activity and clinical intervention. These are reviewed by Silver et al. (1977) who also described the isolation of 30 different bacterial species associated with post-toothbrushing transient bacteraemia, including a high proportion of characteristically oral species, especially *Streptococcus mitis* (*S.mitis*) More recent studies have found oral taxa, such as *Actinomyces* spp, *Streptococcus* spp, *A. actinomycetemcomitans, P. gingivalis, Micromonas micros*, and *S. viridans* (Fowler and Gupta, 2005; Kinane et al., 2005; Gaetti-Jardim et al., 2009; Tomas et al., 2012; Zhang et al., 2013; Horliana et al., 2014). Silver et al. (1977) showed that levels of bacteraemia are dependent on the extent of inflammation and, interestingly, that even minimal levels of gingivitis produced low levels of bacteraemia. Forner et al. (2006) assessed bacteraemia with respect to the extent of gingivitis and periodontal disease and showed moderately positive correlations with *Streptococcus*, the mostly commonly detected genera being *S. mitis*, *S. oralis*, and *S. sanguis*. A systematic review of bacteraemia following daily oral activities (Tomas et al., 2012) concluded that high levels of plaque and gingival indices were associated with significantly increased bacteraemia following toothbrushing; however, the relationship between the extent and duration of bacteraemia with gingival health and bacterial load is not clear.

Early studies showing that blood was not sterile used metabolic measurements (Tedeschi et al., 1969) or lysis-filtration (Heimdahl et al., 1990) where blood cells are lysed under sterile conditions, and bacteria are filtered onto a membrane and cultured for identification under aerobic and anaerobic conditions. Since then, firstly by PCR (Nikkari et al., 2001; McLaughlin et al., 2002) and then NGS, multiple studies have confirmed the presence of bacterial DNA in blood (Amar et al., 2013; Rajendhran et al., 2013; Dinakaran et al., 2014; Paisse et al., 2016; Gosiewski et al., 2017; Li et al., 2018). Blood fractionation kits are often used in conjunction with phenol-chloroform-isoamyl alcohol (PCI) extraction in these studies providing total DNA (and RNA) as template material for amplicon generation. Other systems such as the Phusion Blood Direct kit (Thermo Fisher Scientific) can be used to generate amplicons directly from whole blood (Whittle et al., 2018). Paisse et al. (2016) provided high resolution metagenomic bacterial population profiles using total DNA extracted from red blood cells, buffy coat, and plasma from healthy, young (21 years average) individuals. These profiles were dominated by Proteobacteria (80.4–87.4%), represented mostly by Alphaproteobacteria and various Gammaproteobacteria, with much lower levels of Actinobacteria (6.7–10.0%), Firmicutes (3.0–6.4%), and Bacteroidetes (2.5–3.4%). Studies investigating blood dysbiosis associated with various conditions suggest large variability in blood-borne bacterial populations (Castillo et al., 2019). For instance, in a pancreatitis study (Li et al., 2018), Proteobacteria were dominant with up to 87% of all species classified as gut or faecal-derived (consistent with Paisse et al.). A sepsis study, however, gave Actinobacteria at 70% followed by Proteobacteria at 16% (Gosiewski et al., 2017). One study showed Actinobacteria as dominant in health, but, by contrast Proteobacteria was dominant in disease (Dinakaran et al., 2014). A circulating microbiome study of rheumatoid arthritis (RA) subjects (Hammad et al., 2019) showed that all samples (healthy controls and RA) contained 45.8% Proteobacteria and 31% Firmicutes. Paisse et al. (2016) suggested that high levels of bacterial-derived DNA, measured at 4 X 10^7^ 16S rRNA gene sequences (corresponding to 10^6^–10^7^ bacterial genomes) per ml, are present in blood as cell-free or immune processed fragments.

Despite such high levels of immune-processed bacterial DNA, these types of study are clearly able to differentiate between healthy and dysbiotic blood microbiomes and have provided a wealth of information on both possible healthy blood microbiomes and disease-associated dysbiosis (Lelouvier et al., 2016), but they do not necessarily reflect viable bacterial populations present at the time of sampling. Whittle et al. (2018), in order to address this issue, carried out 16S DNA-based NGS, RNA-based NGS and classical culturing in parallel. Their DNA-based data was consistent with Paisse et al. (2016) with Proteobacteria at 88%, Actinobacteria 7.8%, Firmicutes 3.5%, and Bacteriodetes 0.1% for control samples, although, at the genus level *Achromobacter* was predominant (51%), not *Sphingmonas*. RNA profiles were reasonably consistent with this at the phylum level, but Firmicutes were higher at 19.5% for controls and were consistent with another RNA-based study (Olde Loohuis et al., 2018). Bacterial culture data were deemed overly affected by skin-derived contamination, but demonstrated that neither total DNA or RNA-based microbiome profiling are necessarily accurate reflections of the active or viable microbiota present.

Next Generation Sequencing (NGS) has been used to characterize transient bacteraemia following dental extraction (Benitez-Paez et al., 2013), but no high resolution NGS-based study of periodontitis-associated bacteraemia exists. Here, a commercially available system (MolYsis Complete5) was used for bacterial DNA extraction from blood in order to compare amplicon libraries from periodontally healthy and severe periodontitis cohorts. MolYsis Complete5 has been shown to successfully enrich for intact bacterial cells from human clinical specimens (Horz et al., 2008) including CSF (Hasan et al., 2016), synovial fluid (Thoendel et al., 2016), and urine and blood (Benitez-Paez et al., 2013; Gyarmati et al., 2015; Gyarmati et al., 2016).

This system partially emulates lysis-filtration by purifying bacterial genomic DNA from intact bacterial cells but, instead of the need to culture for taxonomic characterisation, enough purified bacterial genomic DNA is delivered for NGS. Blood represents a unique niche with respect to the characterisation of the microbiome due to its atopobiotic derivation from multiple other source niches with high residual levels of cell-free and immune-processed microbial DNA as a result. Here, we utilized MolYsis technology in order to address this issue and provide blood-borne bacterial population profiles more reflective of viable microbiota present at time of sampling. This was assessed in the context of a metagenomic comparison of bacterial populations in blood from periodontally healthy and severe periodontitis cohorts, in order to describe to the fullest extent possible, bacteraemia resulting from gum disease. Since infective endocarditis and rheumatoid arthritis are associated with the localized presence of oral species, the question arises as to whether persistent, untreated periodontitis is associated with an increased presence of orally derived intact bacteria in addition to the transient increases associated with oral activity already documented.

## Materials and Methods

### Study Design and Participant Details

Participants aged between 19 and 74 years, attending the Bristol Dental Hospital, were recruited to take part in this study during 2018. Patients referred to a specialist Periodontal Clinic for diagnosis of periodontal disease (PD), were assessed by a Basic Periodontal Examination (BPE) (BSP, 2019). Participants deemed PD positive had generalized active periodontitis (Papapanou et al., 2018) with a minimum of four sextants in the mouth scoring 4 on the BPE score, accompanied by signs of generalized inflammation with bleeding on probing.

To be included as controls, the participants’ dental health adhered to the following criteria: BPE scores of 0, demonstrating pristine periodontal health, or with a maximum of 2 sextants with a BPE of 1, or 1 sextant scoring 2 with all other areas scoring 0, representing a patient who has a relatively healthy mouth and no periodontal disease (Lang and Bartold, 2018). Participants with bleeding on probing in less than 10% of probed gingival sites with periodontal pockets ≤3mm were deemed periodontally healthy and were included as controls. Controls (n = 22), mean age ± SD 40 ± 13 years; 17F/5M and age matched patients with periodontitis (n = 18) (mean age ± SD; 46 ± 15 years; 14F/4M). Full subject profiles including BPE scores are shown in **Table 1**.

**Table 1:** Cohort description.

Control Samples								Periodontitis Samples							
Sample	Age	Race	Gender	Smoker?	BPE scores			Sample	Age	Race	Gender	Smoker?	BPE scores		
85	31	Mixed	M	No	0	0	0	84	42	White	F	No	4	3	4
					0	0	0						4	4	(-)
87	25	White	M	No	0	0	0	86	28	White	F	No	3	3	4
					0	1	0						4	4	4
90	22	White	F	No	0	0	0	88	49	White	F	No	4	4	4
					0	1	0						3	4	3
93	34	White	F	No	0	0	0	89	53	White	M	No	4	2	3
					1	0	0						4	4	4
94	21	Asian British	F	No	0	0	1	91	38	White	M	No	4	3	4
					0	1	0						4	3	4
95	21	Asian British	F	No	0	0	0	92	75	Asian British	M	No	(-)	4	4
					0	1	1						4	4	(-)
96	62	White	M	No	1	0	0	97	73	White	M	No	4	4	4
					0	1	0						4	4	4
105	39	White	F	No	0	0	0	98	48	White	F	No	4	4	4
					0	2	0						(-)	4	(-)
108	28	White	F	No	0	0	0	99	26	White	F	No	4	4	4
					0	0	0						3	2	4
110	30	White	F	No	1	0	0	100	31	White	F	No	4	4	4
					0	0	0						4	4	4
111	51	White	F	No	0	0	1	101	60	Black	F	No	4	4	4
					0	0	0						4	3	4
113	36	White	F	No	0	0	0	102	47	White	F	No	4	1	4
					0	1	0						4	4	1
114	42	White	F	No	0	0	0	103	37	White	F	No	4	4	4
					0	1	0						2	1	4
115	56	Asian British	M	No	0	0	0	104	52	White	F	No	4	4	4
					1	0	1						(-)	4	(-)
116	36	White	F	No	1	0	1	106	69	Mixed	F	No	4	4	4
					0	2	0						4	4	4
117	61	White	F	No	0	0	0	107	34	White	F	No	4	4	4
					0	1	0						4	3	4
118	46	White	F	No	1	0	0	109	48	White	F	No	4	4	4
					0	0	1						4	4	4
119	41	White	F	No	0	0	0	112	29	White	F	No	4	4	4
					0	2	0						4	2	4
120	54	White	F	No	1	0	0								
					0	1	0								
121	61	White	F	No	0	0	0								
					0	0	0								
122	44	White	M	No	0	0	0								
					0	0	0								
123	38	White	F	No	0	0	0								
					0	0	0								
Total number of samples = 22								Total number of samples = 18							
Average age = 40								Average age = 47							
								(-) means that there is 1 or no teeth present in that sextant of the mouth.							

### Standard Protocol Approvals, Registrations, and Patient Consent

This study was approved by the National Research Ethics Service (NRES) Southwest Cornwall Plymouth Research Ethics Committee (Approval 13/SW/0272). Written informed consent was obtained from all participants who donated samples for this study.

### Sample Collection

Blood samples were taken not less than two hours after normal daily oral activity and food consumption to avoid transient effects. Venous blood samples were collected in Vacutainer EDTA Blood Collection Tubes (BD, Plymouth, UK) by clinically qualified team members and placed on ice until DNA extraction (within 6 h of collection).

### DNA Extraction

DNA from intact bacterial cells was extracted from 0.5 ml of whole blood using the MolYsis Complete5 system (Molzym GmbH Bremen, Germany), which purifies only genomic DNA from intact bacterial cells, and is designed for Gram-negative and Gram-positive bacteria. Bacterial types detected using MolYsis Complete5 are listed in the manual (https://www.vhbio.com/wp-content/uploads/2017/07/MolYsis_Complete5_V3.0.pdf). DNA extraction was carried out according to the manufacturers’ directions. Briefly: blood cells were lysed with a proprietary chaotropic lysis buffer (containing guanidinium hydrochloride) and host DNA degraded with DNAse. Cell debris and intact bacterial cells were sedimented at 12,000 g with a bench-top centrifuge and the supernatant discarded. The pellet was washed in reducing conditions and bacterial cells treated with proprietary *BugLysis* reagent and digested with proteinase K. Lysate enriched for DNA from intact bacterial cells was then extracted using the Molzym CCT column-based DNA purification system.

Total DNA was extracted from 0.5 ml of whole blood with 0.5 ml of phenol: chloroform: isoamyl alcohol (PCI), in the proportions 25:24:1, equilibrated in T.E. buffer (10 mM Tris pH 8.0, 1 mM EDTA, Sigma Aldrich, St. Louis, Missouri, United States) and DNA ethanol precipitated with 2.5 volumes of ethanol in the presence of 0.2 M NaCl at -20°C overnight. After sedimentation at 17,000 g for 10 min the DNA pellet was washed with 70% ethanol and air-dried before being dissolved in 50 µl of T.E. buffer. The entire procedure was carried out under sterile conditions in a laminar-flow hood.

### DNA Quantification

Initial DNA concentrations were obtained by A260/280 absorption using a Nanodrop spectrophotometer (ThermoFisher Scientific, Waltham, MA, USA). Samples had a A260/280 ratio of between 2 and 1.8. Double-stranded DNA concentrations were determined fluorometrically using a QuantiFluor^®^ dsDNA System (Promega, Madison, Wisconsin, USA) and a FLUOstar Optima microplate reader (BMG Lab Tech, Offenburg, Germany).

### Real-Time PCR

Real-time PCR conditions and cycle parameters were based upon those described by Nadkarni et al. (2002) (Nadkarni et al., 2002) with the following changes: In a 20 µl reaction forward and reverse primers were added to a final concentration of 500 nM along with 100 nM fluorogenic probe and 1 X TaqPath qPCR mastermix (ThermoFisher Scientific). Cycle parameters were: 5 min at 95°C followed by 40 cycles of 30 s at 95°C, then 40 s at 60°C. PCR was performed in a StepOneplus Real-Time PCR system using StepOne software v2.3 (ThermoFisher Scientific). For total bacterial measurements standard curves used a gel-purified and quantified (QuantiFluor) PCR product generated using the same primers and *Escherichia coli* (*E. coli*) DHα1B (K12-derived) genomic DNA template. Standard curve data was always generated on the same PCR plate as sample data from the same mastermix. Primers were the universal 16S rRNA gene variable region 3–4 primers described by Nadkarni et al.: 5’-TCCTACGGGAGGCAGCAGT-3’ (forward, Tm, 59 ± 4°C) and 5’-GGACTACCAGGGTATCTAATCCTGTT-3’ (reverse, Tm, 58 ± 1°C) used in combination with the probe (6-FAM)-5’-CGTATTACCGCGGCTGCTGGCAC-3’-(TAMRA) (Tm, 69 ± 9°C).

Standard curves for streptococcal quantification were derived using a synthetic reference product generated by overlap extension (in a standard single cycle PCR reaction as described below) of 2 oligonucleotides that, when combined, contain the sequence 5’TGAGAGTGGAAAGTTCACACTGTGACGGTATCTTACCAGAAAGGGACGGCTAACTACGTGCCAGCAGCCGCGGTAATACGTAGGTCCCGAGCGTTGTCCGGATTTATTGGGCGTAAAGCGAGCGCAGGCGGTTAGATAAGTCTGAAGTTAAAGGCTGTGGCTTAACCATAGTA CGCTTTGGAAACTGTTTA-3’ which is the exact sequence of the amplicon for *S. mitis* (NR_116207.1) generated by the variable region 3–4 primers used here. (g)*Streptococcus*-specific primers: 5’-TGAGAGTGGAAAGTTCACACTG-3 (forward) and 5’-TAAACAGTTTCCAAAGCGT

ACTAT-3’ (reverse) were designed using the BlastN (blast.ncbi.nlm.nih.gov) (Coordinators, 2018) sequence similarity search suite and the multiple sequence alignment tool Clustal Ω (Madeira et al., 2019) and used in combination with the same probe. These were used under the same conditions (as above) along with the same universal V3–V4 probe.

### 16S Amplicon Libraries

The universal rRNA gene V3-V4 primers described above (Nadkarni et al., 2002) were adapted by the addition of 5’ 8 base pair index extensions and used to generate amplicons under the following conditions: 200 ng of purified template DNA was combined with indexed forward and reverse primers at a final concentration of 1 µM each, dNTPS (ThermoFisher Scientific) at 200 nM each final concentration, 2.5U of GoTaq DNA polymerase (Promega) with 1 X Green GoTaq reaction buffer in a volume of 50 µl. PCR cycle parameters for NGS amplicon generation were: 5 min 95°C, followed by 38 cycles of 30 s at 95°C, 30 s at 65°C, 40 s at 72°C, and then 7 min at 72°C.

Amplicon purification, quantification of DNA, library preparation, sequencing, and data analysis were carried out by Novogene Co Ltd. Beijing, China. Amplicon products were mixed in equi-density ratios and purified with a Qiagen Gel Extraction Kit (Qiagen, Hilden, Germany). The libraries were generated with NEBNext^®^ UltraTM DNA Library Prep Kit for Illumina and quantified *via* Qubit and qPCR, for analysis by the Illumina 2 x 250bp paired end platform (Illumina Inc. San Diego, CA 92122 USA).

### Data Processing

Paired-end reads (PE) were merged using FLASH (V1.2.7, http://ccb.jhu.edu/software/FLASH/) (Magoc and Salzberg, 2011) and subjected to quality filtering with specified parameters (Bokulich et al., 2013) using the Qiime (V1.7.0, http://qiime.org/scripts/split_libraries_fastq.html) (Caporaso et al., 2010) quality control process. The tags were compared with the reference database (Golddatabase, http://drive5.com/uchime/uchime_download.html) using UCHIME algorithm (UCHIMEAlgorithm, http://www.drive5.com/usearch/manual/uchime_algo.html) (Edgar et al., 2011) to detect chimera sequences (http://www.drive5.com/usearch/manual/chimera_formation.html) which were removed (Haas et al., 2011).

### Operational Taxonomic Unit (OTU) Cluster and Species Annotation

Sequence analysis was performed by UPARSE software (UPARSE v7.0.1001 http://drive5.com/uparse/) (Edgar, 2013) using all the effective tags. Sequences with ≥97% similarity were assigned to the same OTUs. For each representative sequence Mothur was used against the SILVA SSU rRNA database (http://www.arb-silva.de/) (Wang et al., 2007) for annotation at each taxonomic rank (Threshold: 0.8~1) (Quast et al., 2013). The multiple sequence alignment tool, MUSCLE (Edgar, 2004) (Version 3.8.31, http://www.drive5.com/muscle/) was used to obtain the phylogenetic relationship of all OTU representative sequences. OTU abundance information was normalized by subsampling using a standard sequence number corresponding to the sample with the least sequences (WB122C: 92,953). Subsequent analysis of alpha diversity and beta diversity, phylogenetic tree construction and downstream statistical analysis were all performed with this output normalized data.

The Human Oral Database (HOMD; homd.org) (Escapa et al., 2018) was used to assign potentially orally derived taxa. This was compared to Source Tracker2 (Knights et al., 2011) analysis using the source sample training data https://gmrepo.humangut.info/data/project/PRJEB6092 (faecal) and https://www.ncbi.nlm.nih.gov/bioproject/PRJNA419453 (subgingival plaque). In order to do this DADA2 (Callahan et al., 2016) was used to merge paired end reads, de-noise, and remove chimeras. Resulting feature tables were de-novo clustered into operational taxonomic units (OTUs) at a 95% identity level. OTUs from “source” samples could be used to train Source Tracker2 to calculate the proportion of likely sources for our “sink” test blood samples.

### Alpha Diversity

Indices of alpha diversity including Chao1, Shannon, and Simpson were calculated with QIIME (Version 1.7.0) and displayed with R software (Version 2.15.3).

### Beta Diversity and Statistical Analysis

Unweighted and Weighted Unifrac phylogenetic distance pairwise matrices of dissimilarity (Lozupone and Knight, 2005; Lozupone et al., 2007; Lozupone et al., 2011) were calculated by QIIME software (Version 1.7.0) and visualized with Principal Coordinate Analysis (PCoA) using R software (Version 2.15.3) and by Unweighted Pair-group Method with Arithmetic Means (UPGMA) hierarchical clustering using QIIME software (Version 1.7.0). Principal Component Analysis (PCA) used the FactoMineR package and ggplot2 package in R software (Version 2.15.3). LEfSe analysis was conducted by LEfSe software (Segata et al., 2011). Metastats was calculated by R software. P-values were calculated by the method of permutation test and corrected for False Discovery Rate (q-values) by the method of Benjamini and Hochberg (White et al., 2009).

## Results

### DNA Yields and Amplicon Generation

The MolYsis Complete5 system yielded on average 1.5 μg of bacterial DNA from 0.5 ml of whole blood from all samples. Two hundred ng of each sample was used for amplicon generation with 35 cycles in a single-stage PCR reaction. Samples 85C, 116C, 120C, and 86P did not yield adequate amplicon to pass pre-NGS quality control. Compared with MolYsis extraction, PCI extraction of total DNA gave adequate levels of bacterial 16S rRNA gene amplicon in 10 samples (out of 40), only 4 of which (3 healthy controls and 1 periodontal patient) were of adequate yield to pass pre-NGS quality control. PCI extraction of total nucleic acid from 0.5 ml of whole blood was used to facilitate a direct comparison with that extracted from the same volume of whole blood using MolYsis Complete5 and does not represent an optimized method for total DNA extraction from blood using PCI. MolYsis products are designed to be completely bacterial DNA-free. This was reflected in no-template PCR controls, where DNA-free MolYsis water was added to DNA spin-columns instead of sample, and the resulting eluate used as template for NGS amplicon generation, which was negative.

### Sequence Data Quality

Sequence read depth and data quality are shown in **Supplementary Table 1** where MolYsis and PCI data are compared alongside alpha diversity indices. Sequence read statistics are displayed in graphical format in **Supplementary Figure 1**. Molysis samples on average yielded 375,558 250 bp paired-end raw tags, 70% of which were effective, detecting on average 203 OTUs (clustered at 97%) per sample. PCI-extracted samples yielded on average 208,334 paired-end tags, of which 70% were effective and detected 193 different OTUs. Alpha rarefaction analysis is displayed in **Supplementary Figure 2**. Sequence data is available at GenBank with Bioproject accession number PRJNA659735.

### Taxa Abundance

#### MolYsis Extracted

**Figure 1:**
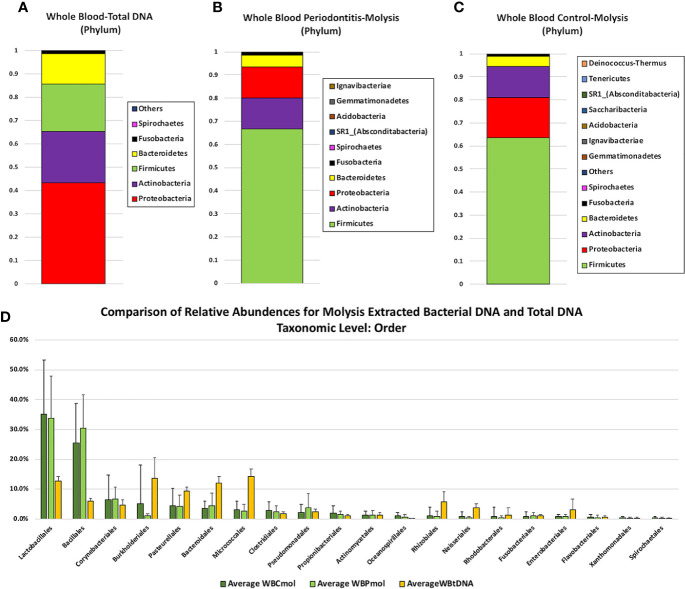
Comparison of phylum level relative abundance profiles from **(A)** total DNA samples extracted from whole blood by PCI (n = 4), **(B)** Molysis-extracted DNA from whole blood from control patients (WBC) (n = 19), and **(C)** Molysis-extracted DNA from blood from patients with periodontitis (WBP) (n = 17).The OTU representing 3 members of the *Streptococcus mitis* group is designated SMG-subgroup. Panel 1**(D)** compares average percentage composition profiles for total DNA samples versus MolYsis-extracted samples at the Order level.

**Figure 2:**
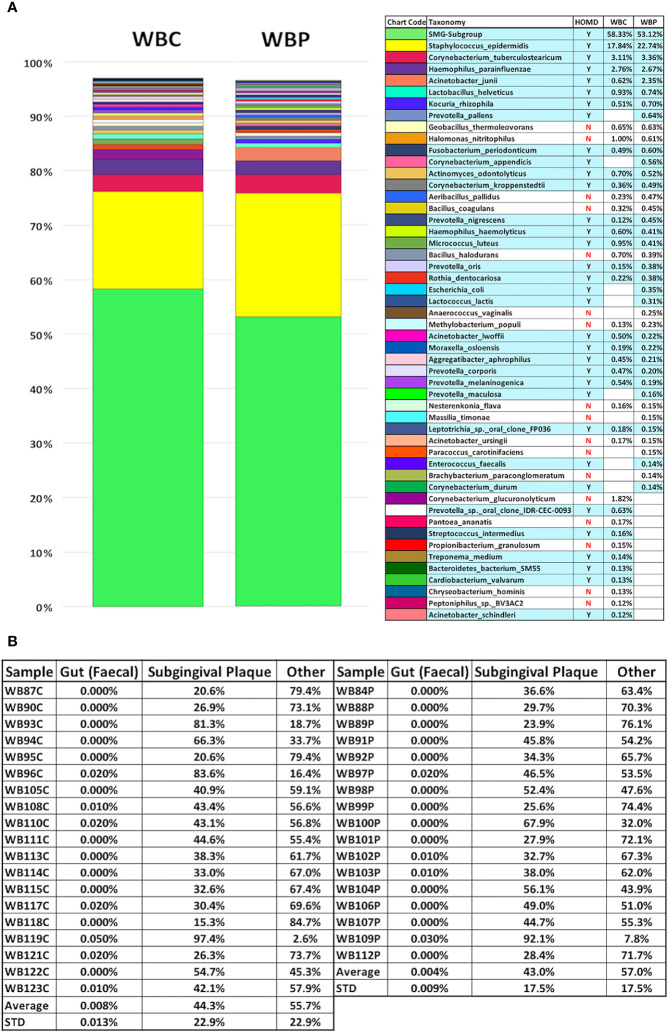
Percentage composition of the 40 most abundant bacterial species in whole blood from the periodontally healthy (WBC) and severe periodontitis (WBP) groups displayed with potentially orally-derived taxa [found in the Human Oral Microbiome Database(homd.org)] indicated by y/n and highlighted in blue **(A)** and Source Tracker2 analysis using faecal and subgingival training datasets **(B)**.

Full taxonomic abundance profiles are shown in **Supplementary Tables 2**–**7**. Phylum level relative abundances for MolYsis extracted samples and total DNA (PCI-extracted) are shown in **Figures 1A–C**. Firmicutes was the largest component in MolYsis samples: 63.5% in periodontally healthy whole blood controls (WBC) and 66.7% in whole blood from severe periodontitis cases (WBP). Proteobacteria was the second largest in WBC at 17.6% and 13.3% in WBP. Others were as follows: Actinobacteria: 13.3% (WBC) and 13.4% (WBP); Bacteroidetes: 4.3% (WBC and 5.1% (WBP); Fusobacteria: 0.8% (WBC) and 1.2% (WBP); Spirochaetes: 0.4% (WBC) and 0.2% (WBP); SR1 was only found in WBP at 0.1%. One hundred fifty different species were detected in WBC and 149 in WBP using the MolYsis system. Relative percentage abundances for WBC and WBP at the species level are shown in **Figure 2**. The main streptococcal taxon in these data represents a subset of the *S. mitis* group, including *infantis*, *oralis* and *mitis*, since variable regions 3 and 4 of the 16S rRNA gene do not differentiate between them at the species level. This taxon is designated as *S. mitis* group-subgroup (SMG-subgroup) in this text. SMG-subgroup represented 58.3% in WBC and 53.1% of total bacterial sequence reads in WBP, and *Staphylococcus epidermidis (S. epidermis*) 17.8% in WBC and 22.7% in WBP.

There were only two taxa that showed significantly different levels between WBC and WBP and both were minor components: (p) Saccharibacteria (q value 0.002), an oral taxon which was not present in WBP and (p) Proteobacteria; (c) Deltaproteobacteria; (o) Myxococcales (q value 0.03), higher in WBC.

#### PCI-Extracted

By comparison, for PCI-extracted total DNA samples, the major components at the phylum level were Proteobacteria (43%), Actinobacteria (22%), Firmicutes (20.4%), Bacteroidetes (13.0%), Fusobacteria (1.1%), and Spirochaetes (0.2%) (**Figure 1**). PCI-extracted samples were compared with Molysis samples at the order level in **Figure 1**. The greatest differences were seen in Lactobacillales (represented primarily by SMG-subgroup) and Bacillales (almost entirely *S. epidermidis*) with higher levels in MolYsis samples. Burholderiales, Pasturellales, Bacteroidales and Micrococales were generally higher in total DNA samples.

### Alpha and Beta-Diversity

**Figure 3:**
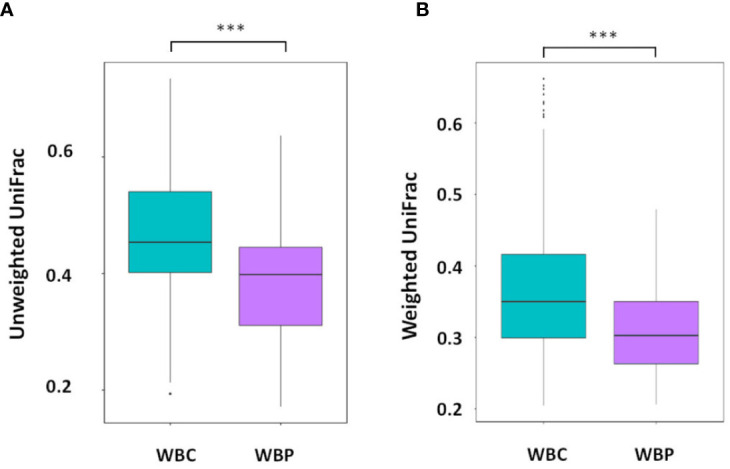
Beta diversity: **(A)** Unweighted and **(B)** weighted UniFrac distances with significant differences between WBC and WBP, P value 0 (Wilcox). ***P < 0.001.

**Figure 4:**
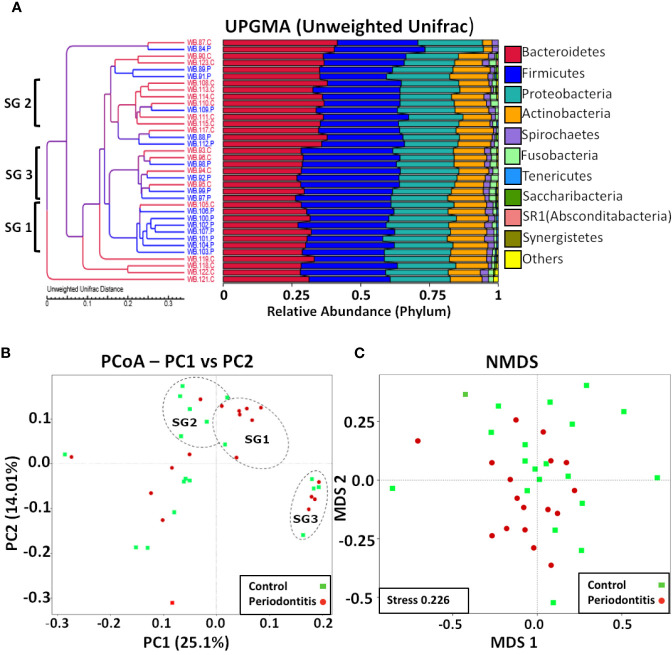
Multivariate analyses of blood bacterial populations. **(A)** UPGMA of unweighted UniFrac distances showing percentage composition at the phylum level with three possible clusters suggested by brackets. **(B)**. PCoA of unweighted UniFrac distances with healthy samples shown in green and periodontitis samples in red and possible clusters (SG1, SG2, and SG3) suggested by UPGMA indicated (for descriptive purposes only) with dashed outlines. **(C)** NMDS analysis showing possible partial partitioning according to health and periodontitis.

Although there were no significant differences in percentage abundance for any major taxonomic component or in alpha diversity indices for health versus periodontitis in whole blood (**Supplementary Table 1**), both unweighted UniFrac (P value 0.0001-Wilcox) and weighted Unifrac values (P value 6.8e^-11^-Wilcox) showed a significant difference in beta diversity (**Figures 3A, B**). Hierarchical clustering by UPGMA (**Figure 4**) suggested partial clustering into subgroups according to periodontitis (SG1) and periodontally healthy (SG2) with the corresponding positions of these clusters in PCoA (4B) outlined by dashed lines for descriptive purposes only. Non-Metric Multi-Dimensional Scaling (NMDS) may indicate some overall partial separation according to periodontitis and controls (stress 0.226) (**Figure 4**).

### Oral Bacterial Species

Of the 150 species, found here, in blood, 52% were oral in origin as defined by the HOMD. It should be noted, however, that *S. epidermidis*, the second largest component, is included as oral for this estimate as it is part of the HOMD but, although it is prevalent in the oral cavity and increased in the periodontal pocket compared to healthy subgingival populations (O’Connor et al., 2018), its oral origin cannot be assumed. For the 40 most abundant species 70% are oral in WBP and 67.5% in WBC which represents over 88% of all bacterial sequences detected in blood. These are listed in **Figure 2** and contain a wide spectrum of commensal oral species and those associated with the subgingival biofilm. Using each entire sample dataset (not just the top 40 according to relative abundance) Source Tracker2 (Knights et al., 2011) was also used to assess orally-derived content (**Figure 2**). This showed that, on average, blood bacterial populations were 44.3%± 22.9% (STD) orally derived in health and 43.0%±17.5% (STD) with periodontitis (see **Figure 2**). This compares to gut-derived content which was seen only in 11 samples and averaged 0.006% overall. The discrepancy between HOMD-based and Source Tracker estimates of oral content may be due to the choice of subgingival training data used in the Source Tracker analysis.

### Real-Time PCR Quantification of Total and Streptococcal 16S rRNA Gene Sequences Using MolYsis-Extracted Template DNA

Only standard curves with R square values of 0.99 and slope values typically -3.473 ± 0.1528 were used. Each measurement was carried out in duplicate and each experiment repeated. Averages of both experiments are shown in **Supplementary Figure 3**. Threshold cycle values (C_t_) for “no template controls” (NTC) for total bacteria measurements ranged from 33.7 to 35.8 and defined the sensitivity of the experiment. Most measured C_t_ values were within 5 C_t_ units of the NTC and, therefore, are only included here as estimates. We estimate that, on average 1 ml of healthy blood contained 4,446 ± 3,111 16S sequences which equates to 889 bacterial cells, assuming an average 16S rRNA gene copy number of 5 (The Ribosomal RNA Database, Schmidt Laboratory, University of Michigan: rrndb.umms.med.umich.edu) (Vetrovsky and Baldrian, 2013) with outliers 118C (average: 27,874 ± 10,184) and 93C (28,852 ± 895) removed. Blood from participants with periodontitis contained 4,567 ± 207 16S sequences per ml, equivalent to 913 bacterial cells per ml on average. There is no significant difference between health and periodontitis. For streptococcal 16S rRNA gene sequences 1 ml of blood from healthy controls contained 2,561 ± 1,657 copies and periodontal blood contained 4,058 ± 4,691 copies (with 93C and 118C removed). Taking into account a 16S rRNA gene copy number of 5.4 for *Streptococcus* this translates to 478 streptococcal cells per ml of healthy blood and 751 in periodontitis but, this does not represent a significant difference between periodontal health and periodontitis for streptococcal sequences. Interestingly, the outlier sample 118C, with a total 16S count of 27,874.9± 10,184.3 also had an unusual relative abundance profile with the opportunistic pathogen Betaproteobacteria; (o)Burkholderiales; (f)*Oxalobacteraceae* representing 54.3% of the total but seen only at no more than 0.05% in all other samples. Outlier sample 93C, as well as having an unusually high total bacteria count also had the highest streptococcal count. See **Supplementary Figure 3**.

## Discussion

Most bacteraemia and blood metagenomic studies employ methods such as PCI-extraction of blood fractions or systems such as the Phusion Blood Direct kit (Thermo Fisher Scientific), both of which generate total DNA (Amar et al., 2013; Rajendhran et al., 2013; Dinakaran et al., 2014; Lelouvier et al., 2016; Paisse et al., 2016; Gosiewski et al., 2017; Li et al., 2018; Castillo et al., 2019; Hammad et al., 2019). These studies tend to show Proteobacteria as the dominant phylum found in blood, but with high variability between studies at lower taxonomic levels, even taking into account disease-associated dysbiosis. The profiles for the limited total DNA cohort shown here in the present study were in general agreement with these studies at the phylum level, with Proteobacteria dominating, although lower than most examples at 43%, and Firmicutes higher at 20.4%.

Paisse et al. (2016), by combining high resolution metagenomic analysis of blood fractions with qPCR measurements of 16S rRNA gene fragment levels showed that the majority of bacterial DNA found in blood is either immune-processed or cell-free fragments. A direct comparison of qPCR measurements and culturing data of post-oral treatment bacteraemia, using total DNA extracted from whole blood (Balejo et al., 2017) may also reflect this issue: total bacterial sequence levels reached 76,442 per ml of blood as measured by qPCR, but only 521 (aerobic) and 782 (anaerobic) from blood cultures after post-dental scaling. Notably, the qPCR-based estimates of bacterial cell numbers shown here, using MolYsis-extracted DNA (on average 889/ml in WBC and 913/ml in WBP), are more consistent with these culturing estimates than with qPCR-based estimates using total DNA. In order to address this issue, Whittle et al. (2018) carried out 16S DNA-based NGS, RNA-based NGS and classical culturing in parallel. Their DNA-based data was consistent with Paisse et al. (2016) with Proteobacteria at 88%, Actinobacteria 7.8%, Firmicutes 3.5%, and Bacteriodetes 0.1% for control samples, although, at the genus level *Achromobacter* was predominant (51%), not *Sphingmonas*. RNA profiles were reasonably consistent with this at the phylum level, but Firmicutes were higher at 19.5% for controls and were consistent with another RNA-based study (Olde Loohuis et al., 2018). Bacterial culture data were deemed overly affected by skin-derived contamination, but demonstrated that neither total DNA or RNA-based microbiome profiling are necessarily accurate reflections of the active or viable microbiota present.

This study took the alternative approach of differential lysis, similar in this respect to the lysis-filtration (Heimdahl et al., 1990), using the MolYsis Complete5 system, that preferentially purifies only genomic DNA from intact bacterial cells. This yielded a strikingly different perspective of the blood microbiome to most other studies, with Firmicutes, rather than Proteobacteria, dominant in both health and disease. Furthermore, bacterial species of oral origin accounted for over 50% of the species found here in blood which is, perhaps, more consistent with earlier lysis-filtration studies with Streptococci predominant (Forner et al., 2006). Unexpectedly, however, this study showed the SMG-subgroup to be the largest component taxon in periodontally healthy controls as well as in severe periodontitis. In addition, not only was there little difference in relative abundances for all major component taxons, there was no significant difference in absolute qPCR measurements of total or streptococcal DNA levels (although there may have been a trend to higher levels in periodontitis). The streptococcal presence seen here, made up mainly of the *mitis* group (58.3% in WBC and 53.1% in WBP), together with *S. epidermidis* (17.8% in WBC and 22.7% in WBP) represented a total relative abundance for the Firmicutes of 76.1% in WBC and 75.8% in WBP, substantially different from previous studies where Proteobacteria dominate and Firmicutes are rarely seen above 15% (for DNA-based studies without MolYsis) and were often absent. A study of transient bacteraemia following dental extraction also used MolYsis for 16S rRNA gene NGS, and compared this with conventional culturing and qPCR measurements of 16S rRNA gene sequences (Benitez-Paez et al., 2013). They also found bacterial abundance profiles were not dominated by Proteobacteria, but were substantially different to the profiles seen here. Most notably, the *Streptococcaceae* were found only at 0.7%, on average. Culturing, however, found that 13 out of 18 isolates were viridans group streptococci (nine *mitis* group and four *salivarius* group), in close agreement with the MolYsis data seen here. A study of bloodstream infections of neutropenic patients also combined 16S rRNA metagenomic analysis using MolYsis-derived samples with culturing (Gyarmati et al., 2015). NGS profiles were dominated by Proteobacteria (average 55.2%) and Firmicutes (average 33.4%), but showed extreme variation in relative proportions of each. Again, culturing results were dominated by viridans group streptococci. PCR-positive and culture-positive samples accounted for only 14.6 and 15.4% of the total, respectively.

### The Blood Microbiome in Health and Periodontitis

Despite significant differences in beta diversity between WBC and WBP there were no significant differences in any major component taxon (above 1%) between bacterial community profiles in blood from participants with no periodontitis and blood from participants with periodontal disease seen here, with only marginal increases in bacterial levels. It may be the case, therefore, that many of the bacteria detected here (at least in WBC) were dormant or non-viable (Potgieter et al., 2015; Kell and Pretorius, 2018): the MolYsis system would not differentiate between these.

Comparison of the blood profiles in this study with the Human Oral Microbiome Database, has revealed an extensive array of bacteria, both commensal and pathogenic, potentially derived from an oral source in not only blood from participants with periodontitis, but also in blood from periodontally healthy controls. Most notably, SMG-subgroup is the most abundant taxon in blood. Furthermore, eight of the top 10 blood species in periodontal healthy participants and six in those with periodontitis were oral. Only two taxa here, (p) Saccharibacteria (formerly TM7) and (c) Deltaproteobacteria (o) Myxococcales were significantly different between periodontal health and individuals exhibiting periodontitis, but were detected at extremely low levels. Saccharibacteria is a ubiquitous and diverse oral phylum, commonly associated with periodontitis (Bor et al., 2019), although, found here only in periodontally healthy controls. There were relatively few species in the 40 most abundant blood taxa that are listed in Sokransky complexes, with red group species such as *P.gingivalis* present at very low levels (0.6% in WBC and 0.8% in WBP), *T.denticola*, present at extremely low levels or, in the case of *T. forsythia*, absent.

Alpha diversity indices between WBC and WBP were not significantly different. However, there were significant differences in phylogenetic profiles, as defined by Unifrac beta diversity distances, between WBC and WBP, with some possible partial clustering suggested by NMDS ordination analysis. Hierarchal branch analysis and PCoA, both indicated some possible clustering with SG1 and SG2 largely corresponding to health and periodontitis, but the nature of SG3 is less clear as it contained equal numbers or periodontal and healthy subjects. A larger study cohort is required to further define these statistically.

### Origins of Blood Microbiomes

Whether there is a healthy, stable microbiome and what its nature is, remains unknown and difficult to study (Castillo et al., 2019). It is clear, however, that due to the unique nature of blood as a tissue, the blood microbiome and its various disease-associated dysbiotic versions, can be described as forming through atopobiosis (Kell and Pretorius, 2015; Potgieter et al., 2015), whereby microbial populations translocate to the blood from other niches. Previously reported blood microbiome profiles are most consistently dominated by Proteobacteria (Amar et al., 2013; Lelouvier et al., 2016; Paisse et al., 2016; Olde Loohuis et al., 2018). This would suggest that the gut is the single most important source of the blood microbiome (Paisse et al., 2016; Castillo et al., 2019). Skin, female reproductive tract, and oral cavity have also been suggested as source niches. Whittle et al. (2018), using *in silico* PCoA comparison between their data and the Human Microbiome Project showed that blood bacterial communities resembled most closely those of the skin and oral cavity. This present study suggested that viable bacterial populations in blood are dominated by species from the oral cavity, with possible contributions from skin and gut. This has implications for the interpretation of studies linking periodontal disease and other inflammatory diseases. Most significantly, a recent consensus report (Sanz et al., 2020) re-emphasized the link between periodontitis and multiple forms of CVD including coronary artery disease (de Boer et al., 2014), cerebrovascular disease (Dietrich et al., 2013), and peripheral artery disease (Yang et al., 2018). Studies of transient bacteraemia following daily oral activities and clinical interventions have shown that oral bacterial species can regularly enter the blood (Tomas et al., 2012), the extent of which is linked to the severity of gingival disease (Balejo et al., 2017). The data shown here is consistent with this but was not the transient result of dental treatment and therefore may represent a continuous, oral bacterial challenge to the circulatory system. Given that 45–50% of the European population are affected by periodontitis (over 11% of which is classified severe periodontitis) (Kassebaum et al., 2014), this represents a considerable risk to cardiovascular health in the population. Furthermore, the levels of oral bacterial DNA seen here in controls, suggests that this risk is not confined to the periodontally challenged.

Rheumatoid arthritis has been linked to dysbiotic microbiomes in several body sites including the mouth (Zhang et al., 2015; Beyer et al., 2018; Lopez-Oliva et al., 2018; Mikuls et al., 2018) and blood (Hammad et al., 2019).

Late-onset Alzheimer’s disease has also been linked to decreased oral health: Measures of decreased gingival health have been associated with cognitive impairment (Stewart et al., 2008; Kamer et al., 2015; Ide et al., 2016; Lee et al., 2017; Holmer et al., 2018) and increased levels of circulating IgG antibodies to oral pathogens such as *A. actinomycetemcomitans*, *P. gingivalis*, and *T. forsythia* have been detected in plasma (Kamer et al., 2009) *and F. nucleatum* and *P. intermedia* in serum (Sparks Stein et al., 2012) of Alzheimer’s patients. Epidemiological studies have also suggested a weak link between periodontal disease and Alzheimer’s disease (Choi et al., 2015; Chen et al., 2017; Choi et al., 2019). The presence of oral bacteria has also been reported in brain tissue from Alzheimer’s patients including spirochetes such as *Treponema species and P. gingivalis* (Riviere et al., 2002; Poole et al., 2013; Miklossy, 2015; Olsen and Singhrao, 2015; Dominy et al., 2019; Emery et al., 2017). These data together suggest periodontitis is a factor in the aetiology of late onset Alzheimer’s disease through direct incursions of periodontal bacterial species into the brain. Consistent with this model, this study provides evidence for a possible blood-borne route of bacterial translocation from the periodontium to the brain.

## Conclusion

The emergence of high throughput sequencing has added greatly to an understanding of the nature and extent of the microbial challenge to the circulatory system and valuable insights into bacteraemia associated with various diseases and a possible healthy blood microbiome. Such studies most commonly use total DNA extraction from blood fractions. These may be limited by the chemical stability of DNA with comparatively high levels of immune-processed and cell-free fragments present even when culturing techniques show little or no viable bacteria. Thus, these types of study do not necessarily represent viable bacterial populations at the time of sampling (Paisse et al., 2016; Whittle et al., 2018). RNA-based sequencing overcomes this problem to some extent (Olde Loohuis et al., 2018; Whittle et al., 2018) but the MolYsis system used here, (similar alternatives may be available) combines the ability to sample possibly viable bacterial cell communities with the sensitivity of 16S rRNA gene NGS. It is also rapid and straightforward to use, requiring only 0.5 ml of blood (or less) and therefore may be a useful additional tool for the measurement and characterisation of bacteraemia. For instance, in its clinical guideline for prophylaxis against infective endocarditis (clinical guideline 64.1, 2015) (Palmer, 2016) the National Institute for Health and Care Excellence highlighted the relative sparsity of data regarding bacteraemia and the need for better ways of measuring it.

This study provides a novel perspective and further understanding of the blood microbiome and microbiota in health and periodontitis with oral bacteria accounting for 88% of bacterial sequence reads in both. This may also provide new insights into the nature of blood-borne bacterial populations important in CVD and other inflammatory diseases such as sporadic late onset Alzheimer’s disease and rheumatoid arthritis.

## Data Availability Statement

This Targeted Locus Study project has been deposited at DDBJ/ENA/GenBank under the accession KEOX000000000. The version described in this paper is the first version, KEOX010000000.

## Ethics Statement

The studies involving human participants were reviewed and approved by the National Research Ethics Service (NRES) Southwest Cornwall Plymouth Research Ethics Committee (Approval 13/SW/0272). The patients/participants provided their written informed consent to participate in this study.

## Author Contributions

DE, SA-B, NW, MD, TC, and JS all helped in the conception of the study and design of the experimental approach. NW, JS, and TC contributed to dental procedures. DE organized collection of data. DE, MD, and TC collected the data, performed laboratory work, and analyzed the output data. AP contributed to data handling and analysis. Ongoing analysis of data was discussed by all participants. DE, SA-B, NW, MD, and TC all contributed to the first draft of the manuscript. All authors contributed to the article and approved the submitted version.

## Funding

We received a donation from GSK as part of their disinterested support into our work on bacteraemia and Alzheimer’s disease. The funder was not involved in the study design, collection, analysis, interpretation of data, the writing of this article or the decision to submit it for publication.

## Conflict of Interest

The authors declare that the research was conducted in the absence of any commercial or financial relationships that could be construed as a potential conflict of interest.

## Supplementary Material

Supplementary Figure 1 Sequencing read statistics.

Supplementary Figure 2 Alpha-rarefaction Analysis.

Supplementary Figure 3 Real-Time PCR Quantification of Total 16S RNA Gene Sequencing.

Supplementary Table 1 Sequence read statistics and alpha diversity indices.

Supplementary Table 2 Relative abundance (Phylum).

Supplementary Table 3 Relative Abundance (Class).

Supplementary Table 4 Relative Abundance (Order).

Supplementary Table 5 Relative Abundance (Family).

Supplementary Table 6 Relative Abundance (Genus).

Supplementary Table 7 Relative Abundance (Species).
