# Supplementary material for: Comparison of Blood Bacterial Communities in Periodontal Health and Periodontal Disease
Source: Front Cell Infect Microbiol. 2021 Jan 5;10:577485. doi: 10.3389/fcimb.2020.577485 (PMC7813997; doi:10.3389/fcimb.2020.577485)
Supplement: Supplementary Figure 1 — Sequencing read statistics. [file DataSheet_1.zip › Supplementary Figure 3.PPTX]

## Slide 1
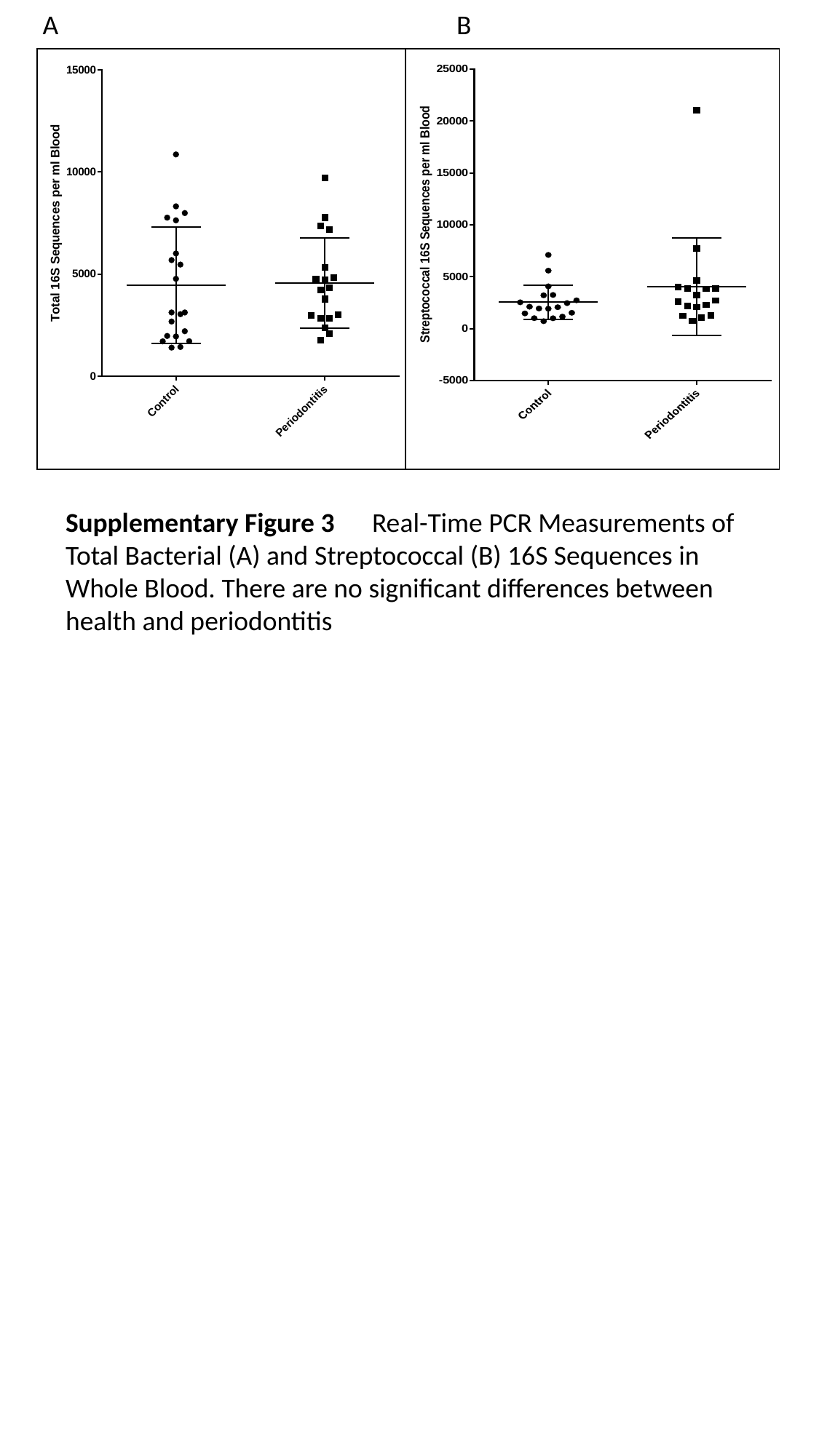

A
B
Supplementary Figure 3 Real-Time PCR Measurements of Total Bacterial (A) and Streptococcal (B) 16S Sequences in Whole Blood. There are no significant differences between health and periodontitis
