# Supplementary figures and images for: Comparison of Blood Bacterial Communities in Periodontal Health and Periodontal Disease
Source: Front Cell Infect Microbiol. 2021 Jan 5;10:577485. doi: 10.3389/fcimb.2020.577485 (PMC7813997; doi:10.3389/fcimb.2020.577485)

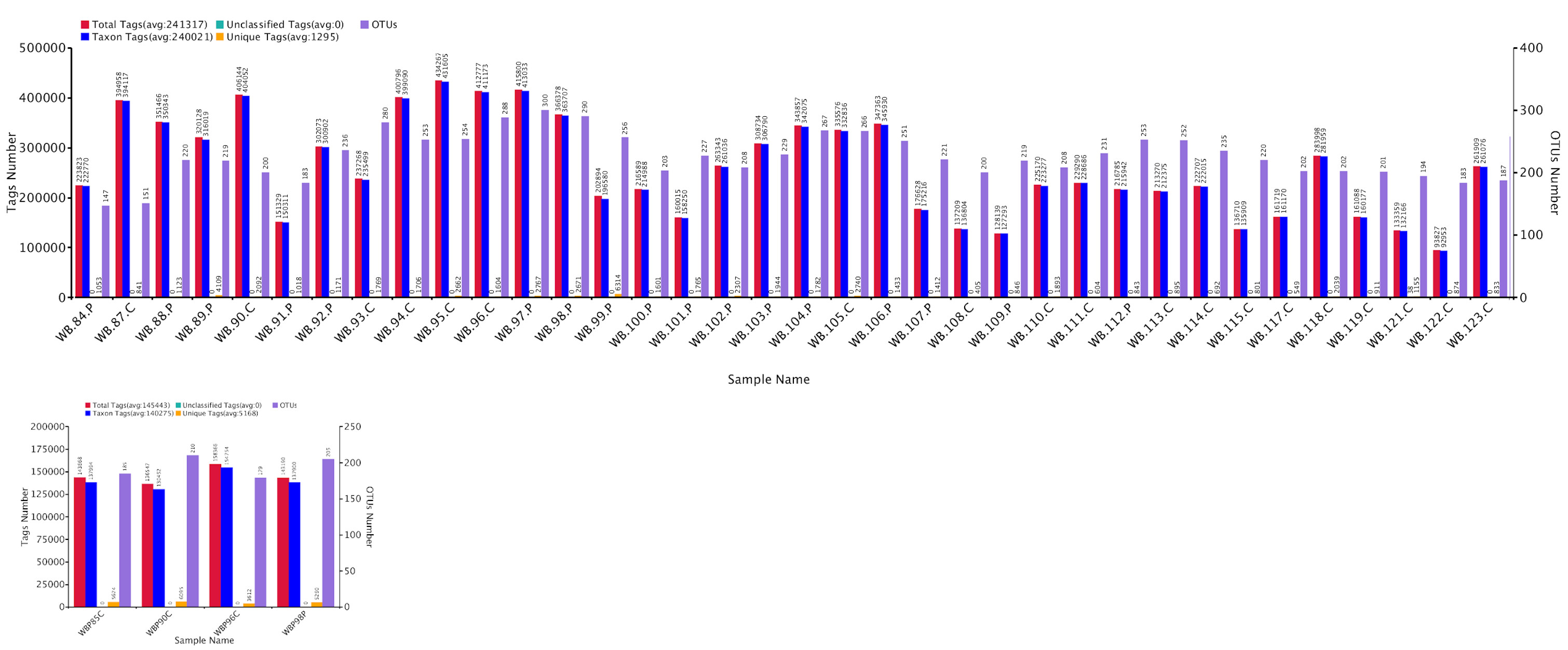

Supplement: Supplementary Figure 1 — Sequencing read statistics. [file DataSheet_1.zip › Supplementary Figure 1.JPEG]
